# Anatomical Distribution of the Superior and Inferior Labial Arteries: A Cadaveric, Systematic Review, and Meta-analysis Study

**DOI:** 10.1007/s00266-026-06037-1

**Published:** 2026-06-30

**Authors:** Gisele Laynes Milla, Hellen Cristina Silva dos Santos, Maria Clara de Sousa, Ingrid de Sousa, Felipe Vitorino, Guilherme Xavier Jorge, Aline Coelho Quezadas, Josué Júnior Araújo Pierote, Renato Assis Machado

**Affiliations:** 1 Master Program, School of Dentistry, Ingá University Center, Maringá, Paraná Brazil; 2 School of Dentistry, São Judas Tadeu University, São Paulo, São Paulo Brazil; 3 Instituto Orofacial das Américas, Piracicaba, São Paulo Brazil; 4 Graduate Program in Oral Biology, School of Dentistry, University of Campinas (FOP-UNICAMP), Piracicaba, São Paulo Brazil; 5 Department of Oral Biology, School of Dentistry, State University of Campinas, Piracicaba, São Paulo 13414-018 Brazil

**Keywords:** Labial artery, Superior labial artery, Inferior labial artery, Cadaveric study, Systematic review, Meta-analysis, Lip anatomy

## Abstract

**Background:**

Precise knowledge of the anatomical course of the superior and inferior labial arteries is essential for ensuring the safety of surgical interventions and minimally invasive esthetic procedures involving the lips. However, despite their growing clinical importance, the available anatomical evidence remains heterogeneous and fragmented, which limits the development of consistent safety guidelines.

**Objective:**

To evaluate the anatomical distribution of the superior and inferior labial arteries in relation to the orbicularis oris muscle by integrating findings from a Brazilian cadaveric anatomical investigation with evidence synthesized through a systematic review and meta-analysis.

**Methods:**

The cadaveric component was performed using preserved Brazilian specimens and standardized anatomical landmarks, dividing the lips into midline and paramedian regions located 1 cm lateral to the midline. The position of the superior and inferior labial arteries relative to the orbicularis oris muscle was classified as submucosal, intramuscular, or subcutaneous. In parallel, a systematic review and meta-analysis were conducted according to PRISMA guidelines, with electronic databases searched without date restrictions for studies reporting the anatomical location of the labial arteries. Eligible studies were quantitatively synthesized using fixed- or random-effects models depending on the degree of heterogeneity.

**Results:**

Four studies met the eligibility criteria for quantitative synthesis. The pooled analysis demonstrated that the superior labial artery in the upper lip was most frequently located in the submucosal plane (33.3–53.3%), followed by an intramuscular course (40.0–46.7%), whereas a subcutaneous position was uncommon (0.0–26.7%). In the lower lip, the inferior labial artery showed greater variability, with intramuscular positioning predominating in median regions (73.3%) and submucosal positioning ranging from 13.3 to 53.3%, while subcutaneous localization remained rare. Regional and sex-related differences contributed to moderate-to-high heterogeneity across the analyses. The cadaveric findings were consistent with the meta-analytic results, confirming the predominance of submucosal and intramuscular arterial courses, a consistent midline anastomosis, and increased positional variability in paramedian regions.

**Conclusions:**

The superior and inferior labial arteries demonstrate variable anatomical distribution patterns, most commonly occupying submucosal and intramuscular planes, while subcutaneous courses are uncommon. By integrating a Brazilian cadaveric study with systematic review and meta-analysis, this investigation provides comprehensive anatomical evidence with direct clinical implications for improving the safety of lip surgery and minimally invasive esthetic procedures, emphasizing that neither midline nor paramedian regions can be considered entirely risk-free.

**No Level Assigned:**

This journal requires that authors assign a level of evidence to each submission to which Evidence-Based Medicine rankings are applicable. This excludes Review Articles, Book Reviews, and manuscripts that concern Basic Science, Animal Studies, Cadaver Studies, and Experimental Studies. For a full description of these Evidence-Based Medicine ratings, please refer to the Table of Contents or the online Instructions to Authors https://link.springer.com/journal/00266.

**Supplementary Information:**

The online version contains supplementary material available at 10.1007/s00266-026-06037-1.

## Introduction

Procedures involving the lips, including reconstructive surgery and minimally invasive esthetic treatments, have increased substantially over the past decade. In particular, the widespread use of hyaluronic acid fillers for lip augmentation and rejuvenation has intensified concerns regarding vascular safety in the perioral region [1, 2]. Although generally considered safe, inadvertent vascular injury during lip procedures may lead to severe complications, such as tissue necrosis, embolization, and, in rare cases, visual loss [1, 3].

The superior and inferior labial arteries, both branches of the facial artery, represent the principal vascular supply of the upper and lower lips, respectively. These vessels exhibit marked anatomical variability in their origin, course, and depth in relation to the orbicularis oris muscle [4, 5]. Previous anatomical investigations have demonstrated that the labial arteries may follow submucosal, intramuscular, or subcutaneous courses, with clinically relevant differences between midline and paramedian regions of the lips [1].

Several cadaveric and imaging-based studies have described aspects of labial artery anatomy, often emphasizing surgical reconstruction, flap design, or the safety of esthetic injections [3, 6–8]. Despite these contributions, most available studies are limited by small sample sizes, methodological heterogeneity, or restricted regional analyses, frequently focusing on a single lip region or arterial segment. Furthermore, although anatomical variability is widely acknowledged, quantitative synthesis of the existing evidence remains scarce, limiting the development of evidence-based safety recommendations.

Importantly, most anatomical data on the labial arteries derive from Caucasian and East Asian populations, with limited representation of highly admixed populations. To date, no Brazilian anatomical study has systematically evaluated the position of the superior and inferior labial arteries in relation to standardized anatomical landmarks, despite Brazil’s well-documented genetic heterogeneity and its potential influence on vascular anatomy. The inclusion of a Brazilian cadaveric investigation, therefore, represents a relevant and novel contribution to the existing literature.

Therefore, the aim of the present study was to systematically review the available literature, quantitatively synthesize anatomical distribution patterns through meta-analysis, and complement these findings with a standardized Brazilian cadaveric study, thereby providing clinically relevant anatomical evidence to guide safer lip surgery and minimally invasive esthetic procedures.

## Materials and Methods

### Study Design

This investigation consisted of three integrated components: (1) an original cadaveric anatomical study, (2) a systematic review of the literature, and (3) a meta-analysis of eligible studies. The systematic review and meta-analysis were conducted in accordance with the Preferred Reporting Items for Systematic Reviews and Meta-analyses (PRISMA) statement [9, 10].

### Cadaveric Study

**Fig. 1:**
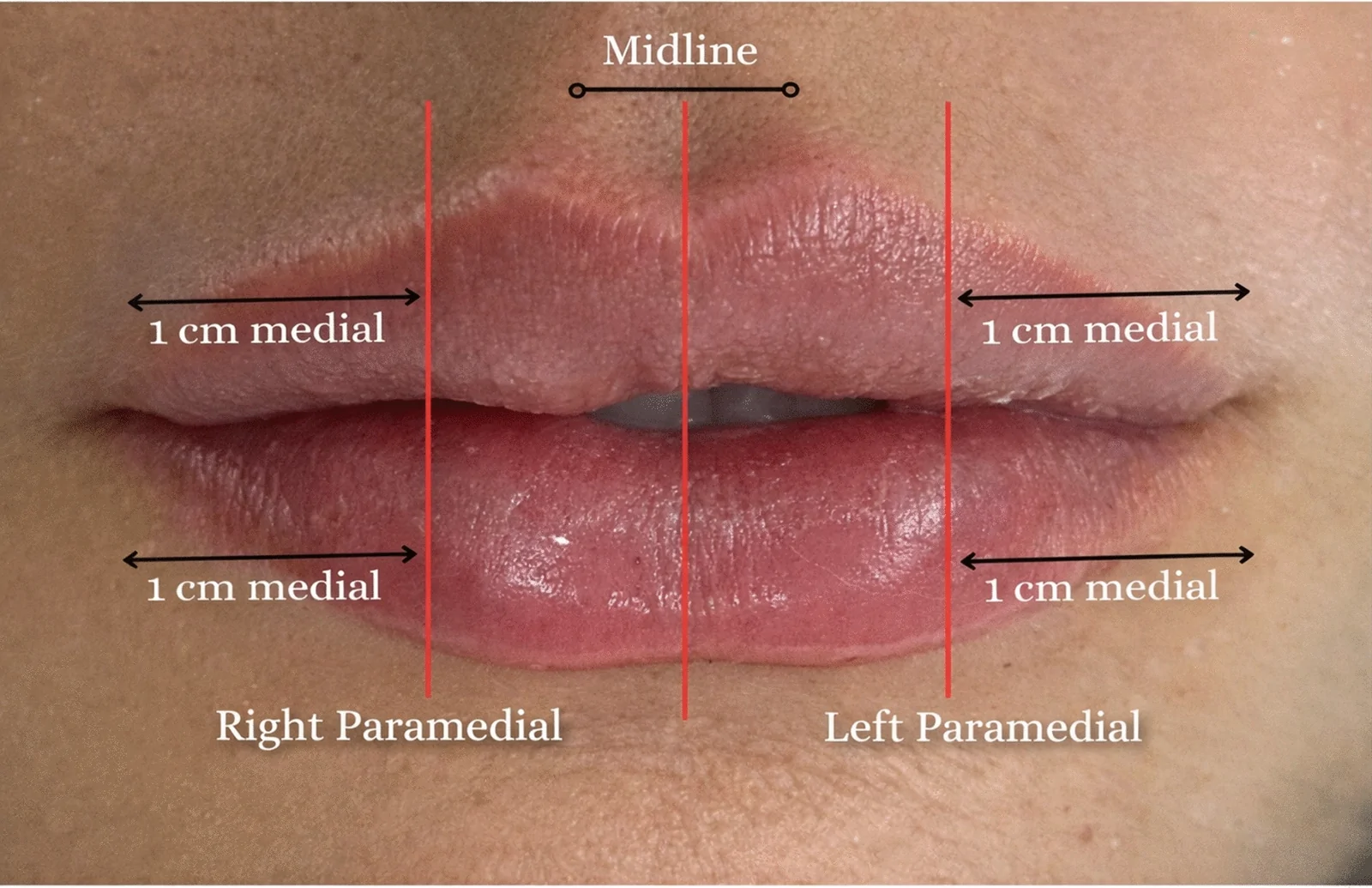
Standardized anatomical reference points used for lip assessment. The midline is identified as the central vertical reference, with the right and left paramedian regions defined bilaterally. Measurements were performed at 1 cm medial to each paramedian line on both the upper and lower lips, ensuring consistent and reproducible spatial analysis across subjects

The cadaveric component of the study was conducted using 15 preserved human cadaveric specimens obtained from the university. All procedures were performed in accordance with Brazilian regulations governing the use of human cadaveric material for research purposes and were submitted to and approved by the institutional ethics committee (CAAE: 78714224.8.0000.0089). Anatomical dissection was performed using standardized vertical incisions measuring 3 cm in length in both the upper and lower lips. Incisions were made at three predefined anatomical locations: the midline, 1 cm medial to the left oral commissure (left paramedian), and 1 cm medial to the right oral commissure (right paramedian) (Fig. 1). Careful layer-by-layer dissection was carried out to identify the main trunk of the superior and inferior labial arteries. The position of each artery was documented in relation to the orbicularis oris muscle and classified as submucosal, intramuscular, or subcutaneous. Specimens in which the arterial position relative to the muscle could not be clearly identified were excluded from the analysis. All findings were recorded photographically and systematically tabulated to allow descriptive comparison with the results of the systematic review and meta-analysis.

### Systematic Review Protocol and Eligibility Criteria

The eligibility criteria were defined according to the Population, Intervention, Comparison, Outcomes, and Study design (PICOS) framework. The research question focused on the anatomical distribution of the superior and inferior labial arteries in humans. Accordingly, studies were considered eligible if they met the following criteria: (P) Population—human subjects, including cadaveric specimens or in vivo anatomical assessments; (I) Intervention/Exposure—anatomical identification and evaluation of the superior and/or inferior labial arteries; (C) Comparison—not applicable; (O) Outcomes—description of the arterial position relative to the orbicularis oris muscle, classified as submucosal, intramuscular, or subcutaneous; and (S) Study design—original anatomical studies, including cadaveric dissections and imaging-based investigations.

Studies were excluded if they: (i) did not allow identification of the spatial relationship between the labial arteries and the orbicularis oris muscle; (ii) involved non-human specimens; or (iii) were secondary publications, including narrative or systematic reviews, letters, conference abstracts, case reports, case series, expert opinions, or editorials.

### Information Sources and Search Strategy

A comprehensive search was performed in PubMed (https://pubmed.ncbi.nlm.nih.gov), Scopus (https://www.scopus.com), Embase (https://www.embase.com), Web of Science (https://www.webofscience.com/wos/woscc/basic-search), Cochrane Library (https://www.cochranelibrary.com), and LIVIVO (https://www.livivo.de) databases. Gray literature was searched using ProQuest. The search included all articles published up to December 2025. Search terms combined controlled vocabulary and free-text keywords related to sex, cadaveric specimens, and labial artery anatomy. All retrieved records were imported into the Rayyan reference management software, where duplicate entries were identified and removed [11]. The reference lists of all included studies were manually reviewed to identify additional relevant publications that met the inclusion criteria. The complete search strategy and database-specific queries are detailed in Supplementary Table 1.

### Study Selection

**Fig. 2:**
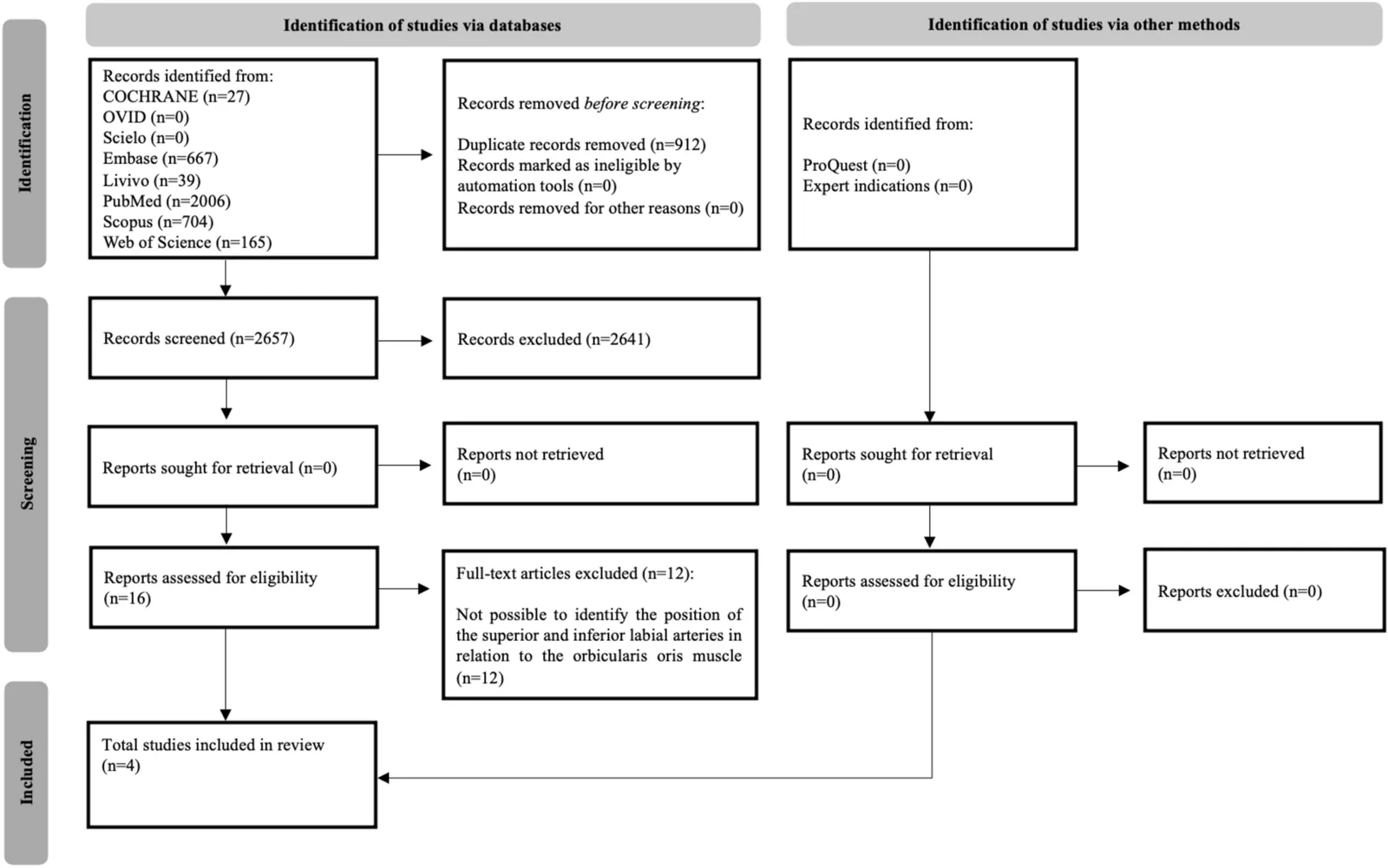
Flow diagram of literature search and selection criteria adapted from Preferred Reporting Items for Systematic Reviews and Meta-Analyses (PRISMA)

The study selection process was conducted in two distinct phases. In phase 1, two reviewers independently screened the titles and abstracts of all retrieved records to identify potentially eligible studies using the Rayyan software [11]. Studies that clearly did not meet the predefined inclusion criteria were excluded at this stage. When uncertainties or disagreements arose regarding eligibility, a third reviewer was consulted to reach a consensus decision. In phase 2, the same two reviewers independently assessed the full-text versions of all potentially eligible articles to confirm the availability of relevant anatomical data on the positional relationship of the superior and inferior labial arteries to the orbicularis oris muscle. The same eligibility criteria were applied during full-text evaluation. Reference lists of all included studies were subsequently reviewed by all three authors to identify additional relevant publications. Any discrepancies occurring at either phase were resolved through discussion and consensus among the reviewers. The final selection consisted exclusively of full-text articles that fulfilled all predetermined inclusion criteria. The overall study selection process is summarized in a PRISMA flow diagram (Fig. 2).

### Data Extraction

Data extraction was independently performed by two reviewers using a standardized data collection form. The accuracy and completeness of the extracted data were subsequently verified through cross-checking by a third reviewer. Any discrepancies were resolved through discussion and consensus among the three authors. For each included study, the following information was extracted: country of origin, study design, sample size, sex distribution, anatomical methodology (cadaveric dissection), lip region evaluated (upper or lower lip; median or paramedian), and the anatomical position of the superior and/or inferior labial arteries relative to the orbicularis oris muscle, classified as submucosal, intramuscular, or subcutaneous. When relevant data were incomplete or unclear, attempts were made to contact the corresponding authors to obtain additional information necessary for quantitative synthesis.

### Risk of Bias and Certainty of Evidence

Risk of bias was independently assessed by two reviewers using the Joanna Briggs Institute (JBI) Critical Appraisal Tools for cohort studies. Prior to the appraisal process, all authors discussed and agreed upon the scoring criteria to ensure consistency in assessment. Any disagreements between reviewers were resolved through discussion, with consultation of a third reviewer when necessary. Studies were categorized according to their overall risk of bias as low (> 70% of “yes” responses), moderate (50–69%), or high (≤ 49%). Detailed risk-of-bias assessments are presented in Supplementary Table 2.

The overall certainty of the evidence was evaluated using the Grading of Recommendations, Assessment, Development, and Evaluation (GRADE) approach, considering domains of risk of bias, inconsistency, indirectness, and imprecision. The GRADEpro Guideline Development Tool was used to support this process. The certainty of evidence for each outcome was classified as high, moderate, low, or very low, and the results are summarized in Supplementary Table 3.

### Meta-analysis

Meta-analyses were performed using the *metabin* function in the R meta-package (R software version 6.5-0, JJ Allaire, Boston, Massachusetts, USA). Odds ratios (ORs) with 95% confidence intervals (CIs) were calculated for each arterial plane. A random-effects model was applied when heterogeneity exceeded 50% (*I*^2 ^> 50%); otherwise, a fixed-effect model was used.

## Results

### Cadaveric Study

**Fig. 3:**
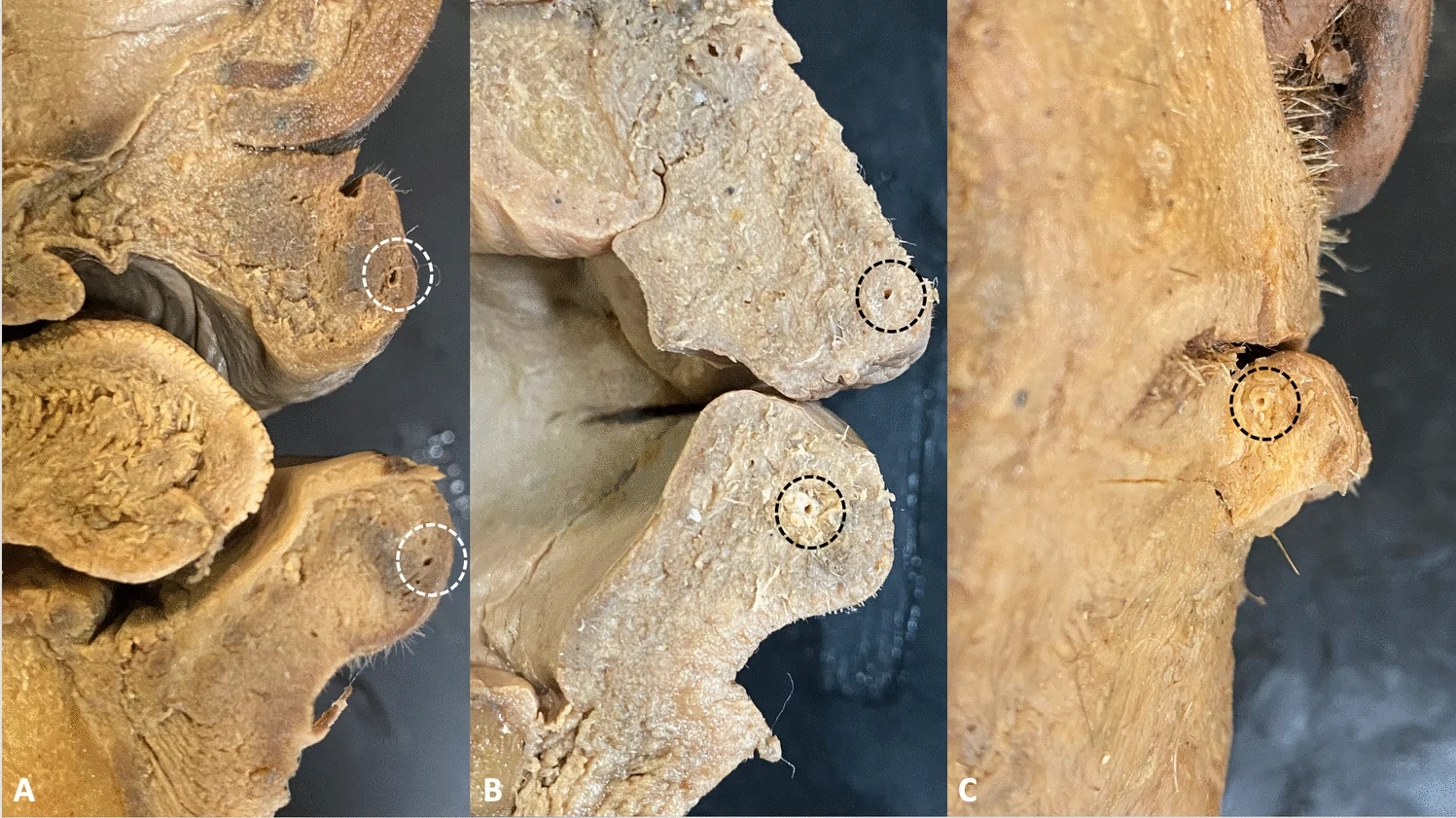
Anatomical dissection of the position of the superior and inferior labial arteries within the lips: **A** Subcutaneous in the upper and lower lips (i.e., between the skin and the orbicularis oris muscle); **B** Subcutaneous in the upper lip and intramuscular in the lower lip (i.e., between the superficial and deep layers of the orbicularis oris muscle); **C** Submucosal in the lower lip (i.e., between the oral mucosa and the orbicularis oris muscle)

The findings of the original cadaveric dissections are summarized in Table 1 and schematically illustrated in Fig. 3. The superior and inferior labial arteries were identified bilaterally in all specimens. In the upper lip, the superior labial artery most frequently coursed within the submucosal plane, particularly in the paramedian regions, accounting for 46.7% of cases on the right and 46.7% on the left, while in the midline, the submucosal position was observed in 33.3% of specimens. An intramuscular course was also common, ranging from 40.0% to 46.7%, whereas a subcutaneous position was less frequent (6.7–26.7%), being most prevalent at the midline. Notably, a consistent midline anastomotic pattern of the superior labial artery was observed across specimens. In the lower lip, the inferior labial artery demonstrated greater positional variability. The intramuscular plane predominated, particularly in the median (73.3%), followed by submucosal positioning (13.3–53.3%). A subcutaneous course remained uncommon (13.3%) and was absent in the midline. Overall, the inferior labial artery exhibited a more heterogeneous anatomical distribution than the superior labial artery, reflecting its well-documented variability in origin and depth.

**Table 1:** Distribution of the location of the superior and inferior labial arteries in the upper and lower lips

Location	Upper lip			Lower lip		
	Right paramedian	Median	Left paramedian	Right paramedian	Median	Left paramedian
Submucosal	7 (46.7%)	5 (33.3%)	7 (46.7%)	8 (53.3%)	2 (13.3%)	6 (40.0%)
Intramuscular	6 (40.0%)	6 (40.0%)	7 (46.7%)	7 (46.7%)	11 (73.3%)	7 (46.7%)
Subcutaneous	2 (13.3%)	4 (26.7%)	1 (6.7%)	0 (0.0%)	2 (13.3%)	2 (13.3%)

### Study Selection to Systematic Review

The study selection process is illustrated in the PRISMA 2020 flow diagram (Fig. 2). A total of 2657 records were screened after the removal of duplicates. Sixteen full-text articles were assessed for eligibility, of which 12 were excluded due to insufficient anatomical detail regarding the spatial relationship of the superior and inferior labial arteries to the orbicularis oris muscle (Supplementary Table 4). Ultimately, four studies fulfilled the inclusion criteria and were incorporated into the qualitative synthesis and quantitative meta-analysis.

### Characteristics of Included Studies

All eligible studies were observational cohort investigations with an anatomical focus, predominantly based on cadaveric dissections, with one study additionally employing ultrasound imaging to assess arterial depth. The studies were conducted across multiple countries, including the USA, Germany, Austria, Canada, the Commonwealth of Dominica, and Brazil, and comprised specimens of predominantly Caucasian ancestry, with limited representation of individuals of African descent. In total, 276 cadaver heads were analyzed across the included studies, with variable sex distribution. Sample sizes ranged from 9 to 193 specimens, reflecting substantial differences in study scale. Anatomical assessment consistently focused on the superior and/or inferior labial arteries, evaluating their course relative to the orbicularis oris muscle and their spatial distribution within median and paramedian regions of the upper and lower lips. Across studies, arterial positioning was systematically classified into submucosal, intramuscular, and subcutaneous planes, allowing direct comparison of distribution patterns between lip regions, sides, and sexes. Despite methodological heterogeneity in specimen preparation and imaging techniques, all studies reported sufficient anatomical detail to permit pooled quantitative analysis and subgroup comparisons (Table 2).

**Table 2:** Main characteristics of the selected papers

Study	Study design	Country	Ancestry	No. of patients	Sex		Sample	Labial location	Superior lip			Superior lip	Inferior lip			Inferior lip	Method used
					Male	Female			Right paramedian	Median	Left paramedian	Average	Right paramedian	Median	Left paramedian	Average	
Schulte et al. (2009)	Cohort	USA	Caucasian	9	4	5	Total	Submucosal				7.0				8.0	Anatomical dissection
								Intramuscular				2.0				1.0	
								Subcutaneous				0.0				0.0	
Cotofana et al. (2017)	Cohort	Commonwealth of Dominica, Austria, Munich, and Germany	Caucasian	193	84	109	Total	Submucosal	150	149	153	150.7	166	123	163	150.7	Anatomical dissection
								Intramuscular	36	36	30	34.0	23	55	22	33.3	
								Subcutaneous	5	4	6	5.0	2	6	2	3.3	
Cotofana et al. (2020)	Cohort	USA, Canada, and Germany	40 Caucasians and 1 African–American	41	20	21	Total	Submucosal	24	24	26	24.7	22	22	26	23.3	Ultrasound
								Intramuscular	14	17	14	15.0	14	18	13	15.0	
								Subcutaneous	3	0	1	1.3	5	1	2	2.7	
							Male	Submucosal				12.0				12.0	
								Intramuscular				7.0				7.0	
								Subcutaneous				1.0				1.0	
							Female	Submucosal				12.0				12.0	
								Intramuscular				8.0				8.0	
								Subcutaneous				1.0				1.0	
Money et al. (2020)	Cohort	USA	Caucasian	18	9	9	Total	Submucosal	16	17	16	16.3				0.0	Anatomical dissection
								Intramuscular	1	1	1	1.0				0.0	
								Subcutaneous	1	0	1	0.7				0.0	
Our study	Cohort	Brazil	Caucasian and African	15	13	2	Total	Submucosal	7	5	7	6.3	8	2	6	5.3	Anatomical dissection
								Intramuscular	6	6	7	6.3	7	11	7	8.3	
								Subcutaneous	2	4	1	2.3	0	2	2	1.3	
							Male	Submucosal	7	5	6	6.0	7	2	5	4.7	
								Intramuscular	6	5	6	5.7	6	10	6	7.3	
								Subcutaneous	2	3	1	2.0	0	1	2	1.0	
							Female	Submucosal	0	0	1	0.3	1	0	1	0.7	
								Intramuscular	2	1	1	1.3	1	1	1	1.0	
								Subcutaneous	0	1	0	0.3	0	1	0	0.3	

### Systematic Review and Meta-analysis

Pooled analyses demonstrated that the superior labial artery was most frequently located in the submucosal plane across all regions of the upper lip, with a pooled odds ratio (OR) of 0.71 (95% CI: 0.54–0.85) and substantial heterogeneity (*I*^2 ^= 72.6%). This was followed by an intramuscular course (OR 0.22, 95% CI: 0.11–0.37; *I*^2 ^= 68.7%), whereas a subcutaneous position was rare (OR 0.01, 95% CI: 0.00–0.04; *I*^2 ^= 5.4%) (Table 3). A similar overall distribution pattern was observed for the inferior labial artery, although with greater variability. Submucosal positioning remained predominant (OR 0.65, 95% CI: 0.43–0.85; *I*^2 ^= 82.6%), followed by intramuscular localization (OR 0.27, 95% CI: 0.12–0.46; *I*^2 ^= 78.7%). As in the upper lip, a subcutaneous course was uncommon (OR 0.02, 95% CI: 0.00–0.07; *I*^2 ^= 31.9%) (Table 3).

**Table 3:** Distribution patterns of labial artery locations in the lips

	Number of studies	Number of events	Number of observations	OR (95% CI)	*p*-value	*Z* effect	*I*^2^	Analysis model
Superior lip								
Right paramedian								
Submucosal	4	197	267	0.69 (0.52–0.85)	0.005	12.90	76.8%	Random effect
Intramuscular	4	57	267	0.22 (0.10–0.37)	0.01	10.08	70.3%	Random effect
Subcutaneous	4	11	267	0.02 (0.00–0.55)	0.16	5.14	41.6%	Common effect
Median								
Submucosal	4	195	267	0.68 (0.42–0.90)	0.0001	20.73	85.5%	Random effect
Intramuscular	4	60	267	0.24 (0.09–0.42)	0.002	14.50	79.3%	Random effect
Subcutaneous	4	8	267	0.02 (0.00–0.14)	0.01	11.15	73.1%	Random effect
Left paramedian								
Submucosal	4	202	267	0.71 (0.54–0.86)	0.008	11.63	74.2%	Random effect
Intramuscular	4	52	267	0.22 (0.08–0.41)	0.002	14.48	79.3%	Random effect
Subcutaneous	4	9	267	0.02 (0.00–0.04)	0.70	1.42	0.0%	Common effect
Total								
Submucosal	5	205	276	0.71 (0.54–0.85)	0.005	14.59	72.6%	Random effect
Intramuscular	5	58.3	276	0.22 (0.11–0.37)	0.01	12.76	68.7%	Random effect
Subcutaneous	5	9.3	276	0.01 (0.00–0.04)	0.37	4.23	5.4%	Common effect
Inferior lip								
Right paramedian								
Submucosal	3	196	249	0.67 (0.41–0.88)	<0.0001	23.44	91.5%	Random effect
Intramuscular	3	43	249	0.25 (0.08–0.46)	0.0006	14.95	86.6%	Random effect
Subcutaneous	3	7	249	0.02 (0.00–0.12)	0.01	9.07	77.9%	Random effect
Median								
Submucosal	3	147	249	0.44 (0.17–0.74)	0.0003	16.04	87.5%	Random effect
Intramuscular	3	84	249	0.45 (0.22–0.70)	0.001	13.80	85.5%	Random effect
Subcutaneous	3	9	249	0.02 (0.00–0.05)	0.24	2.82	29.0%	Common effect
Left paramedian								
Submucosal	3	195	249	0.66 (0.39–0.88)	<0.0001	18.92	89.4%	Random effect
Intramuscular	3	42	249	0.26 (0.08–0.49)	0.0003	16.51	87.9%	Random effect
Subcutaneous	3	6	249	0.03 (0.00–0.12)	0.03	6.56	69.5%	Random effect
Total								
Submucosal	4	187.3	258	0.65 (0.43–0.85)	0.0006	17.25	82.6%	Random effect
Intramuscular	4	57.3	258	0.27 (0.12–0.46)	0.002	14.11	78.7%	Random effect
Subcutaneous	4	7.3	258	0.02 (0.00–0.07)	0.22	4.41	31.9%	Common effect

Subgroup analyses revealed regional differences along the lip. In the midline, both superior and inferior labial arteries demonstrated a higher likelihood of a submucosal course (superior lip: OR 0.68, 95% CI: 0.42–0.90; *I*^2 ^= 85.5%; inferior lip: OR 0.44, 95% CI: 0.17–0.74; *I*^2 ^= 87.5%). In contrast, in the paramedian regions (1 cm from the midline), intramuscular positioning was more frequently observed, particularly for the inferior labial artery (right paramedian: OR 0.25, 95% CI: 0.08–0.46; *I*^2 ^= 86.6%) (Table 3).

Sex-stratified analyses demonstrated subtle differences in arterial depth and plane (Table 4). In females, both superior and inferior labial arteries showed a predominance of submucosal positioning (superior lip: OR 0.24, 95% CI: 0.00–0.86; *I*^2 ^= 93.2%; inferior lip: OR 0.27, 95% CI: 0.00–0.84; *I*^2 ^= 91.8%), whereas in males, a relatively higher proportion of intramuscular courses was observed, particularly in the inferior lip (OR 0.40, 95% CI: 0.24–0.57; *I*^2 ^= 0.0%). Subcutaneous localization remained infrequent in both sexes across all analyses.

**Table 4:** Distribution patterns of labial artery locations in the lips according to sex

	Number of studies	Number of events	Number of observations	OR (95% CI)	*p*-value	*Z* effect	*I*^2^	Analysis model
Superior lip								
Female								
Submucosal	2	12.3	36	0.24 (0.00–0.86)	0.0001	14.79	93.2%	Random effect
Intramuscular	2	9.3	36	0.22 (0.01–0.55)	0.04	3.96	74.8%	Random effect
Subcutaneous	2	1.3	36	0.03 (0.00–0.13)	0.77	0.08	0.0%	Common effect
Male								
Submucosal	2	18	35	0.51 (0.31–0.70)	0.25	1.28	21.9%	Common effect
Intramuscular	2	12.7	35	0.36 (0.20–0.53)	0.85	0.03	0.0%	Common effect
Subcutaneous	2	3	35	0.08 (0.00–0.20)	0.41	0.67	0.0%	Common effect
Inferior lip								
Female								
Submucosal	2	12.7	36	0.27 (0.00–0.84)	0.0005	12.22	91.8%	Random effect
Intramuscular	2	9	36	0.21 (0.00–0.56)	0.02	4.76	79.0%	Random effect
Subcutaneous	2	1.3	36	0.03 (0.00–0.13)	0.77	0.08	0.0%	Common effect
Male								
Submucosal	2	16.7	35	0.46 (0.19–0.73)	0.10	2.67	62.6%	Random effect
Intramuscular	2	14.3	35	0.40 (0.24–0.57)	0.43	0.62	0.0%	Common effect
Subcutaneous	2	2	35	0.05 (0.00–0.16)	0.81	0.06	0.0%	Common effect

## Discussion

This study provides an integrated anatomical synthesis of the superior and inferior labial arteries by combining a systematic review, meta-analysis, and original cadaveric investigation. The principal finding is that both arteries most commonly course within the submucosal plane, particularly in the midline region, whereas intramuscular positioning predominates in paramedian areas located 1 cm from the midline. Across all analytical approaches, a subcutaneous arterial course was consistently uncommon. These findings are concordant with classical cadaveric investigations and contemporary imaging-based studies, supporting the reproducibility of these anatomical patterns across different methodologies [1, 4, 5, 12, 13].

The predominance of a submucosal arterial course in the midline has direct clinical implications for both reconstructive surgery and minimally invasive esthetic procedures. Midline lip augmentation, vermilion border injections, and surgical flap design inherently traverse regions of high vascular density, increasing the risk of arterial injury if anatomical variability is not adequately considered [1, 14, 15]. Although paramedian regions have traditionally been regarded as relatively safer zones for filler placement, the present findings demonstrate frequent intramuscular arterial trajectories in these areas, reinforcing that no region of the lip can be considered entirely risk-free.

The meta-analytic results further corroborate earlier observations that the inferior labial artery exhibits greater anatomical variability than its superior counterpart, both in origin and depth [4, 12]. This variability has been attributed to differences in facial artery termination patterns and the presence of accessory branches, such as the labiomental artery, which contribute variably to lower lip perfusion [16, 17]. Such vascular complexity highlights the importance of individualized anatomical consideration when planning lower lip surgery or filler injection.

In addition to regional variability, biological sex emerged as a relevant modifier of labial artery anatomy. Sex-stratified analyses revealed subtle but consistent differences in arterial depth and course, with female specimens showing a higher prevalence of submucosal positioning, whereas male specimens more frequently exhibited intramuscular arterial courses, particularly in the inferior lip and paramedian regions. These findings are consistent with previous anatomical and imaging-based investigations suggesting that sex-related differences in soft tissue thickness, muscle mass, and connective tissue composition may influence vascular depth and trajectory [1, 6, 7, 14]. From a clinical perspective, intramuscular arterial courses may increase susceptibility to vascular injury during deeper injections or surgical dissection, suggesting that sex-specific anatomical considerations may refine risk assessment and procedural planning. Nevertheless, these observations should be interpreted with caution due to limited sample sizes and interstudy heterogeneity, underscoring the need for future investigations with balanced sex representation and standardized methodologies.

Importantly, the original cadaveric component of the present study was conducted in a Brazilian population, characterized by marked genetic heterogeneity resulting from the historical contribution of Caucasian, African, and Amerindian ancestral groups, forming a relatively unique tri-hybrid ancestry pattern [18, 19]. This degree of genetic admixture may contribute to increased anatomical variability, particularly in vascular patterns, and represents a relevant consideration when extrapolating anatomical data derived predominantly from more genetically homogeneous populations. In this context, the Brazilian cadaveric findings add valuable anatomical evidence that enhances the external validity of existing literature and improves applicability to highly admixed populations.

Recent advances in imaging techniques further support the present findings. High-resolution ultrasound and three-dimensional computed tomography angiography studies have demonstrated a predominantly deep-to-superficial trajectory of the superior and inferior labial arteries, with significant interindividual and population-specific variation influenced by sex, age, and ancestry [13, 20]. These imaging-based observations complement cadaveric evidence and underscore the importance of interpreting vascular anatomy as a dynamic and population-dependent feature rather than a fixed anatomical constant.

A major strength of this investigation lies in its integrative design, combining original cadaveric dissections with a systematic review and quantitative meta-analysis. The use of standardized anatomical landmarks—specifically the midline and paramedian regions located 1 cm from the midline—mirrors the methodology of landmark anatomical studies and facilitates direct comparison and external validation of the findings [1, 15]. The strong concordance between the cadaveric results and pooled meta-analytic estimates reinforces the robustness and clinical relevance of the conclusions.

Several limitations should be acknowledged. First, the number of studies eligible for quantitative synthesis was limited, reflecting the scarcity of investigations providing detailed descriptions of arterial depth relative to the orbicularis oris muscle. Second, heterogeneity was moderate to high in several subgroup analyses, likely due to methodological differences, sample size variability, and population-specific anatomical characteristics. Third, although the inclusion of a Brazilian cadaveric sample enhances population diversity, most available studies remain concentrated in Caucasian and East Asian populations, limiting broader generalizability. Finally, cadaveric studies may not fully replicate in vivo conditions, particularly with respect to vessel caliber, collapsibility, and positional changes associated with facial expression and aging.

In summary, this integrative anatomical investigation demonstrates that the superior and inferior labial arteries predominantly follow submucosal and intramuscular courses, with substantial regional, individual, and sex-related variability. The inclusion of a Brazilian cadaveric sample representative of a highly admixed population further strengthens the relevance of these findings. Enhanced awareness of sex- and population-specific vascular patterns may contribute to improved procedural planning, refined injection techniques, and a reduction in potentially severe vascular complications during lip surgery and minimally invasive esthetic interventions.

## Conclusions

The superior and inferior labial arteries demonstrate consistent yet highly variable anatomical distribution patterns, predominantly within submucosal and intramuscular planes, with a subcutaneous course being uncommon. Midline regions exhibit the highest vascular density, whereas paramedian areas show greater variability rather than guaranteed safety. By integrating systematic review, meta-analysis, and original cadaveric dissection, this study provides robust anatomical evidence with direct clinical implications for lip reconstruction and minimally invasive esthetic procedures. A thorough understanding of labial artery anatomy, combined with cautious technique selection, remains essential to minimizing vascular complications.

## Supplementary Information

Below is the link to the electronic supplementary material.

Supplementary file1 (DOCX 15 kb)

Supplementary file2 (DOCX 21 kb)

Supplementary file3 (DOCX 20 kb)

Supplementary file4 (DOCX 17 kb)
