# Supplementary material for: Anatomical Distribution of the Superior and Inferior Labial Arteries: A Cadaveric, Systematic Review, and Meta-analysis Study
Source: Aesthetic Plast Surg. 2026 Jun 30;50(15):6446–56. doi: 10.1007/s00266-026-06037-1 (PMC13493396; doi:10.1007/s00266-026-06037-1)
Supplement: Supplementary file 1 [file 266_2026_6037_MOESM1_ESM.docx]

**Table 1.** Distribution of the location of the superior and inferior labial arteries in the upper and lower lips.

| **Location** | **Upper Lip** | | | | | | | **Lower Lip** | | |
| --- | --- | --- | --- | --- | --- | --- | --- | --- | --- | --- |
|  | **Right**  **Paramedian** | | | **Median** | **Left**  **Paramedian** | | | **Right**  **Paramedian** | **Median** | **Left**  **Paramedian** |
|  |  | | |  |  | | |  |  |  |
| **Submucosal** | 7 (46.7%) | | | 5 (33.3%) | 7 (46.7%) | | | 8 (53.3%) | 2 (13.3%) | 6 (40.0%) |
| **Intramuscular** | | 6 (40.0%) | 6 (40.0%) | | | 7 (46.7%) | 7 (46.7%) | | 11 (73.3%) | 7 (46.7%) |
| **Subcutaneous** | 2 (13.3%) | | | 4 (26.7%) | 1 (6.7%) | | | 0 (0.0%) | 2 (13.3%) | 2 (13.3%) |
