# Supplementary material for: Anatomical Distribution of the Superior and Inferior Labial Arteries: A Cadaveric, Systematic Review, and Meta-analysis Study
Source: Aesthetic Plast Surg. 2026 Jun 30;50(15):6446–56. doi: 10.1007/s00266-026-06037-1 (PMC13493396; doi:10.1007/s00266-026-06037-1)
Supplement: Supplementary file 2 [file 266_2026_6037_MOESM2_ESM.docx]

**Table 2.** Main Characteristics of the selected papers.

| **Study** | **Study Design** | **Country** | **Ancestry** | **No. of patients** | **Sex** | | **Sample** |  | **Superior lip** | | | **Superior lip** | **Inferior lip** | | | **Inferior lip** | **Method used** |
| --- | --- | --- | --- | --- | --- | --- | --- | --- | --- | --- | --- | --- | --- | --- | --- | --- | --- |
|  |  |  |  |  | **Male** | **Female** |  | **Labial location** | **Right paramedian** | **Median** | **Left paramedian** | **Average** | **Right paramedian** | **Median** | **Left paramedian** | **Average** |  |
| Schulte et al. (2009) | Cohort | USA | Caucasian | 9 | 4 | 5 | Total | Submucosal |  |  |  | 7.0 |  |  |  | 8.0 | Anatomical dissection |
|  |  |  |  |  |  |  |  | Intramuscular |  |  |  | 2.0 |  |  |  | 1.0 |  |
|  |  |  |  |  |  |  |  | Subcutaneous |  |  |  | 0.0 |  |  |  | 0.0 |  |
|  |  |  |  |  |  |  |  |  |  |  |  |  |  |  |  |  |  |
| Cotofana et al. (2017) | Cohort | Commonwealth of Dominica, Austria, Munich and Germany | Caucasian | 193 | 84 | 109 | Total | Submucosal | 150 | 149 | 153 | 150.7 | 166 | 123 | 163 | 150.7 | Anatomical dissection |
|  |  |  |  |  |  |  |  | Intramuscular | 36 | 36 | 30 | 34.0 | 23 | 55 | 22 | 33.3 |  |
|  |  |  |  |  |  |  |  | Subcutaneous | 5 | 4 | 6 | 5.0 | 2 | 6 | 2 | 3.3 |  |
|  |  |  |  |  |  |  |  |  |  |  |  |  |  |  |  |  |  |
| Cotofana et al. (2020) | Cohort | USA, Canada and Germany | 40 Caucasians and 1 African-American | 41 | 20 | 21 | Total | Submucosal | 24 | 24 | 26 | 24.7 | 22 | 22 | 26 | 23.3 | Ultrasound |
|  |  |  |  |  |  |  |  | Intramuscular | 14 | 17 | 14 | 15.0 | 14 | 18 | 13 | 15.0 |  |
|  |  |  |  |  |  |  |  | Subcutaneous | 3 | 0 | 1 | 1.3 | 5 | 1 | 2 | 2.7 |  |
|  |  |  |  |  |  |  |  |  |  |  |  |  |  |  |  |  |  |
|  |  |  |  |  |  |  | Male | Submucosal |  |  |  | 12.0 |  |  |  | 12.0 |  |
|  |  |  |  |  |  |  |  | Intramuscular |  |  |  | 7.0 |  |  |  | 7.0 |  |
|  |  |  |  |  |  |  |  | Subcutaneous |  |  |  | 1.0 |  |  |  | 1.0 |  |
|  |  |  |  |  |  |  |  |  |  |  |  |  |  |  |  |  |  |
|  |  |  |  |  |  |  | Female | Submucosal |  |  |  | 12.0 |  |  |  | 12.0 |  |
|  |  |  |  |  |  |  |  | Intramuscular |  |  |  | 8.0 |  |  |  | 8.0 |  |
|  |  |  |  |  |  |  |  | Subcutaneous |  |  |  | 1.0 |  |  |  | 1.0 |  |
|  |  |  |  |  |  |  |  |  |  |  |  |  |  |  |  |  |  |
| Money et al. (2020) | Cohort | USA | Caucasian | 18 | 9 | 9 | Total | Submucosal | 16 | 17 | 16 | 16.3 |  |  |  | 0.0 | Anatomical dissection |
|  |  |  |  |  |  |  |  | Intramuscular | 1 | 1 | 1 | 1.0 |  |  |  | 0.0 |  |
|  |  |  |  |  |  |  |  | Subcutaneous | 1 | 0 | 1 | 0.7 |  |  |  | 0.0 |  |
|  |  |  |  |  |  |  |  |  |  |  |  |  |  |  |  |  |  |
| Our study | Cohort | Brazil | Caucasian and African | 15 | 13 | 2 | Total | Submucosal | 7 | 5 | 7 | 6.3 | 8 | 2 | 6 | 5.3 | Anatomical dissection |
|  |  |  |  |  |  |  |  | Intramuscular | 6 | 6 | 7 | 6.3 | 7 | 11 | 7 | 8.3 |  |
|  |  |  |  |  |  |  |  | Subcutaneous | 2 | 4 | 1 | 2.3 | 0 | 2 | 2 | 1.3 |  |
|  |  |  |  |  |  |  |  |  |  |  |  |  |  |  |  |  |  |
|  |  |  |  |  |  |  | Male | Submucosal | 7 | 5 | 6 | 6.0 | 7 | 2 | 5 | 4.7 |  |
|  |  |  |  |  |  |  |  | Intramuscular | 6 | 5 | 6 | 5.7 | 6 | 10 | 6 | 7.3 |  |
|  |  |  |  |  |  |  |  | Subcutaneous | 2 | 3 | 1 | 2.0 | 0 | 1 | 2 | 1.0 |  |
|  |  |  |  |  |  |  |  |  |  |  |  |  |  |  |  |  |  |
|  |  |  |  |  |  |  | Female | Submucosal | 0 | 0 | 1 | 0.3 | 1 | 0 | 1 | 0.7 |  |
|  |  |  |  |  |  |  |  | Intramuscular | 2 | 1 | 1 | 1.3 | 1 | 1 | 1 | 1.0 |  |
|  |  |  |  |  |  |  |  | Subcutaneous | 0 | 1 | 0 | 0.3 | 0 | 1 | 0 | 0.3 |  |
|  |  |  |  |  |  |  |  |  |  |  |  |  |  |  |  |  |  |
|  |  |  |  |  |  |  |  |  |  |  |  |  |  |  |  |  |  |
|  |  |  |  |  |  |  |  |  |  |  |  |  |  |  |  |  |  |
