# Supplementary material for: Anatomical Distribution of the Superior and Inferior Labial Arteries: A Cadaveric, Systematic Review, and Meta-analysis Study
Source: Aesthetic Plast Surg. 2026 Jun 30;50(15):6446–56. doi: 10.1007/s00266-026-06037-1 (PMC13493396; doi:10.1007/s00266-026-06037-1)
Supplement: Supplementary file 3 [file 266_2026_6037_MOESM3_ESM.docx]

| **Table 3.** Distribution patterns of labial artery locations in the lips. | | | | | |  |  |  |
| --- | --- | --- | --- | --- | --- | --- | --- | --- |
|  | **Number of studies** | **Number of events** | **Number of observations** | **OR (95% CI)** | **p-value** | **Z effect** | **I^2^** | **Analysis model** |
| **Superior lip** |  |  |  |  |  |  |  |  |
| **Right paramedian** |  |  |  |  |  |  |  |  |
| Submucosal | 4 | 197 | 267 | 0.69 (0.52-0.85) | 0.005 | 12.90 | 76.8% | Random effect |
| Intramuscular | 4 | 57 | 267 | 0.22 (0.10-0.37) | 0.01 | 10.08 | 70.3% | Random effect |
| Subcutaneous | 4 | 11 | 267 | 0.02 (0.00-0.55) | 0.16 | 5.14 | 41.6% | Common effect |
| **Median** |  |  |  |  |  |  |  |  |
| Submucosal | 4 | 195 | 267 | 0.68 (0.42-0.90) | 0.0001 | 20.73 | 85.5% | Random effect |
| Intramuscular | 4 | 60 | 267 | 0.24 (0.09-0.42) | 0.002 | 14.50 | 79.3% | Random effect |
| Subcutaneous | 4 | 8 | 267 | 0.02 (0.00-0.14) | 0.01 | 11.15 | 73.1% | Random effect |
| **Left paramedian** |  |  |  |  |  |  |  |  |
| Submucosal | 4 | 202 | 267 | 0.71 (0.54-0.86) | 0.008 | 11.63 | 74.2% | Random effect |
| Intramuscular | 4 | 52 | 267 | 0.22 (0.08-0.41) | 0.002 | 14.48 | 79.3% | Random effect |
| Subcutaneous | 4 | 9 | 267 | 0.02 (0.00-0.04) | 0.70 | 1.42 | 0.0% | Common effect |
| **Total** |  |  |  |  |  |  |  |  |
| Submucosal | 5 | 205 | 276 | 0.71 (0.54-0.85) | 0.005 | 14.59 | 72.6% | Random effect |
| Intramuscular | 5 | 58.3 | 276 | 0.22 (0.11-0.37) | 0.01 | 12.76 | 68.7% | Random effect |
| Subcutaneous | 5 | 9.3 | 276 | 0.01 (0.00-0.04) | 0.37 | 4.23 | 5.4% | Common effect |
| **Inferior lip** |  |  |  |  |  |  |  |  |
| **Right paramedian** |  |  |  |  |  |  |  |  |
| Submucosal | 3 | 196 | 249 | 0.67 (0.41-0.88) | <0.0001 | 23.44 | 91.5% | Random effect |
| Intramuscular | 3 | 43 | 249 | 0.25 (0.08-0.46) | 0.0006 | 14.95 | 86.6% | Random effect |
| Subcutaneous | 3 | 7 | 249 | 0.02 (0.00-0.12) | 0.01 | 9.07 | 77.9% | Random effect |
| **Median** |  |  |  |  |  |  |  |  |
| Submucosal | 3 | 147 | 249 | 0.44 (0.17-0.74) | 0.0003 | 16.04 | 87.5% | Random effect |
| Intramuscular | 3 | 84 | 249 | 0.45 (0.22-0.70) | 0.001 | 13.80 | 85.5% | Random effect |
| Subcutaneous | 3 | 9 | 249 | 0.02 (0.00-0.05) | 0.24 | 2.82 | 29.0% | Common effect |
| **Left paramedian** |  |  |  |  |  |  |  |  |
| Submucosal | 3 | 195 | 249 | 0.66 (0.39-0.88) | <0.0001 | 18.92 | 89.4% | Random effect |
| Intramuscular | 3 | 42 | 249 | 0.26 (0.08-0.49) | 0.0003 | 16.51 | 87.9% | Random effect |
| Subcutaneous | 3 | 6 | 249 | 0.03 (0.00-0.12) | 0.03 | 6.56 | 69.5% | Random effect |
| **Total** |  |  |  |  |  |  |  |  |
| Submucosal | 4 | 187.3 | 258 | 0.65 (0.43-0.85) | 0.0006 | 17.25 | 82.6% | Random effect |
| Intramuscular | 4 | 57.3 | 258 | 0.27 (0.12-0.46) | 0.002 | 14.11 | 78.7% | Random effect |
| Subcutaneous | 4 | 7.3 | 258 | 0.02 (0.00-0.07) | 0.22 | 4.41 | 31.9% | Common effect |
