# Supplementary material for: Anatomical Distribution of the Superior and Inferior Labial Arteries: A Cadaveric, Systematic Review, and Meta-analysis Study
Source: Aesthetic Plast Surg. 2026 Jun 30;50(15):6446–56. doi: 10.1007/s00266-026-06037-1 (PMC13493396; doi:10.1007/s00266-026-06037-1)
Supplement: Supplementary file 4 [file 266_2026_6037_MOESM4_ESM.docx]

| **Table 4.** Distribution patterns of labial artery locations in the lips according to sex. | | | | | |  |  |  |
| --- | --- | --- | --- | --- | --- | --- | --- | --- |
|  | **Number of studies** | **Number of events** | **Number of observations** | **OR (95% CI)** | **p-value** | **Z effect** | **I^2^** | **Analysis model** |
| **Superior lip** |  |  |  |  |  |  |  |  |
| **Female** |  |  |  |  |  |  |  |  |
| Submucosal | 2 | 12.3 | 36 | 0.24 (0.00-0.86) | 0.0001 | 14.79 | 93.2% | Random effect |
| Intramuscular | 2 | 9.3 | 36 | 0.22 (0.01-0.55) | 0.04 | 3.96 | 74.8% | Random effect |
| Subcutaneous | 2 | 1.3 | 36 | 0.03 (0.00-0.13) | 0.77 | 0.08 | 0.0% | Common effect |
| **Male** |  |  |  |  |  |  |  |  |
| Submucosal | 2 | 18 | 35 | 0.51 (0.31-0.70) | 0.25 | 1.28 | 21.9% | Common effect |
| Intramuscular | 2 | 12.7 | 35 | 0.36 (0.20-0.53) | 0.85 | 0.03 | 0.0% | Common effect |
| Subcutaneous | 2 | 3 | 35 | 0.08 (0.00-0.20) | 0.41 | 0.67 | 0.0% | Common effect |
| **Inferior lip** |  |  |  |  |  |  |  |  |
| **Female** |  |  |  |  |  |  |  |  |
| Submucosal | 2 | 12.7 | 36 | 0.27 (0.00-0.84) | 0.0005 | 12.22 | 91.8% | Random effect |
| Intramuscular | 2 | 9 | 36 | 0.21 (0.00-0.56) | 0.02 | 4.76 | 79.0% | Random effect |
| Subcutaneous | 2 | 1.3 | 36 | 0.03 (0.00-0.13) | 0.77 | 0.08 | 0.0% | Common effect |
| **Male** |  |  |  |  |  |  |  |  |
| Submucosal | 2 | 16.7 | 35 | 0.46 (0.19-0.73) | 0.10 | 2.67 | 62.6% | Random effect |
| Intramuscular | 2 | 14.3 | 35 | 0.40 (0.24-0.57) | 0.43 | 0.62 | 0.0% | Common effect |
| Subcutaneous | 2 | 2 | 35 | 0.05 (0.00-0.16) | 0.81 | 0.06 | 0.0% | Common effect |
